# Alcohol intoxication and risk factors in adolescents

**DOI:** 10.1186/1940-0640-8-S1-A80

**Published:** 2013-09-04

**Authors:** Mireille de Visser, Nicolaas van der Lely, Eva van Zanten

**Affiliations:** 1 Department of Pediatric Psychology, Reinier de Graaf Group, Delft, the Netherlands

## 

Although public interventions aimed at alcohol use among adolescents have been proven effective, individual follow-up of adolescents admitted with alcohol intoxication is uncommon. According to the ESPAD study, many European countries have to deal with a high prevalence of alcohol use amongst adolescents. Alcohol use has been associated with ADHD, autism, negative health behaviour, and neurocognitive damage. Parental rules are a major risk factor for alcohol use in adolescents.

To psychosocially screen, educate and follow-up on adolescents admitted with alcohol intoxication.

In January 2009 a protocol was implemented for the follow-up on adolescents admitted with alcohol intoxication. Screening for chronic alcohol abuse, underlying psychological disorders and social problems was done by the pediatrician and child-psychologist. Information on the dangers of alcohol use was given to adolescents and their parents. During 2009 and 2010 a total of 350 children were invited to the program, of which 204 were screened. Of the 16-year-old adolescents 61% stopped drinking, and 88,5% stopped binge-drinking at 6 months follow-up. In 82,5% parents reported to have implemented specific alcohol rules at follow-up. A total of 90% adolescents were screened with behavioral questionnaires, and 41,2% had psychosocial problems. In clinical practice, history taking of these adolescents revealed shocking stories on social, psychological or alcohol related issues. By informing adolescents and their parents during a clinical and outpatient setting the window of opportunity is widened and alcohol use can be decreased. Underlying social and psychological problems are frequent, serious and should be evaluated in adolescents admitted with alcohol intoxication.

